# Ferroptosis inducer erastin sensitizes NSCLC cells to celastrol through activation of the ROS–mitochondrial fission–mitophagy axis

**DOI:** 10.1002/1878-0261.12936

**Published:** 2021-03-17

**Authors:** Ming Liu, Yumei Fan, Danyu Li, Bihui Han, Yanxiu Meng, Fei Chen, Tianchan Liu, Zhiyuan Song, Yu Han, Liying Huang, Yanzhong Chang, Pengxiu Cao, Akira Nakai, Ke Tan

**Affiliations:** ^1^ Key Laboratory of Animal Physiology, Biochemistry and Molecular Biology of Hebei Province College of Life Sciences Hebei Normal University Shijiazhuang China; ^2^ Department of Respiration Langfang Fourth People’s Hospital China; ^3^ Department of Neurosurgery HanDan Central Hospital China; ^4^ Department of Biochemistry and Molecular Biology Yamaguchi University School of Medicine Ube Japan

**Keywords:** autophagy, celastrol, erastin, mitochondrial fission, mitophagy, non‐small‐cell lung cancer

## Abstract

Despite recent progress in non‐small‐cell lung cancer (NSCLC) treatment, treatment outcomes remain poor, mainly because of treatment resistance or toxicity. Erastin is a ferroptosis inducer that has shown promising cytotoxic effects in various types of cancers, including NSCLC. Celastrol is a triterpene extracted from the *Tripterygium wilfordii* that exhibits potential anticancer activity. However, the side effects of celastrol are severe and limit its clinical application. Combination therapy is a promising strategy to overcome the compensatory mechanisms and unwanted off‐target effects. In the present study, we found that erastin synergized with celastrol to induce cell death at nontoxic concentrations. The combined treatment with celastrol and erastin significantly increased reactive oxygen species (ROS) generation, disrupted mitochondrial membrane potential, and promoted mitochondrial fission. Furthermore, cotreatment with erastin and celastrol initiated ATG5/ATG7‐dependent autophagy, PINK1/Parkin‐dependent mitophagy, and the expression of heat shock proteins (HSPs) in an HSF1‐dependent manner. HSF1 knockdown further enhanced cell death *in vitro* and inhibited tumor growth *in vivo*. Our findings indicate that the combination of celastrol with erastin may represent a novel therapeutic regimen for patients with NSCLC and warrants further clinical evaluation.

## Graphical abstract

Combination therapy is a promising strategy for NSCLC to overcome the compensatory mechanisms and unwanted off‐target effects. Here, we found that the combination of celastrol and erastin synergistically inhibited NSCLC cell growth *in vitro* and *in vivo* by inducing ROS generation, mitochondrial dysfunction, mitochondrial fission, and mitophagy. Our data provide a conceptual framework for the development of a novel strategy for combined treatment utilizing erastin and celastrol.

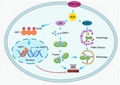

**Abbreviations**
CCK8: Cell Counting Kit‐8
ChIP: chromatin immunoprecipitation
CI: combination index
co‐IP: co‐immunoprecipitation
DRP1: dynamin‐related protein 1
FIS1: mitochondrial fission 1 protein
GCLC: glutamate/cysteine ligase
GSH: glutathione
HGB: hemoglobin
HSF1: heat shock factor 1
HSPs: heat shock proteins
KO: knockout
L‐OPA1: long optic atrophy 1
LYMs: lymphocytes
MDA: malondialdehyde
MFF: mitochondrial fission factor
MFN1: mitofusin 1
NQO1: NAD(P)H‐quinone oxidoreductase 1
NSCLC: non‐small‐cell lung cancer
PI: propidium iodide
Pol II: RNA polymerase II
RBCs: red blood cells
ROS: reactive oxygen species
WBCs: white blood cells

## 1 Introduction

Lung cancer remains the leading cause of cancer‐associated death worldwide, with an estimated 2.1 million cases and 1.8 million deaths in 2018 [1]. Among lung cancer patients, approximately 80–85% are classified as non‐small‐cell lung cancer (NSCLC) according to clinical statistical analyses [2, 3]. The classical treatments for NSCLC consist of surgical resection, radiotherapy, and chemotherapy. Most patients with NSCLC do not receive correct and timely diagnoses until reaching the advanced stages, which include distant metastasis or invasion, thus missing the best time for surgical treatment [2, 3]. At present, the prognosis of NSCLC is still poor, and the 5‐year survival rate is relatively low because of obtrusive drug resistance and potent side effects of chemotherapy [4]. Although immune checkpoint inhibitors targeting PD‐1 and PD‐L1 have been developed, consistent clinical benefits of these immune‐modulating agents are limited to a subset of patients. Therefore, additional effective treatments must be developed through molecular studies to reduce the burden induced by lung cancer.

Erastin is a classical inducer of ferroptosis that has shown promising pharmacologic effects in cancer treatment [5]. Ferroptosis is a newly recognized type of regulated cell death induced by erastin in cancer cells [6]. Ferroptosis is characterized by overwhelming iron accumulation and lipid peroxidation. Erastin is a small molecule that blocks cystine uptake into cells by inhibiting the specific light‐chain subunit of the cystine/glutamate antiporter (SLC7A11 or system Xc^−^). Thus, erastin treatment leads to impaired cellular cystine import, suppressed GSH synthesis, and altered redox status, resulting in lipid peroxidation and ultimately ferroptotic cell death. Recently, an increasing number of studies have revealed a close relationship between ferroptosis and cancer, which has been regarded as a new strategy for developing therapeutic reagents with high therapeutic efficacy in multiple cancers [7, 8].

Celastrol, also known as tripterine, is a natural compound extracted from the traditional Chinese medicinal herb Thunder God Vine (*Tripterygium wilfordii*) [9]. Celastrol has been listed as one of the five traditional medicinal compounds that is most likely to be developed as a modern drug [10]. In recent years, celastrol has attracted considerable interest because of its substantial therapeutic properties in the treatment of multiple diseases, including obesity, rheumatoid arthritis, allergic asthma, systemic lupus erythematosus, lateral sclerosis, and Alzheimer’s disease [11, 12, 13]. However, the most attractive function of celastrol is its potential antitumor effect, which has been demonstrated in a wide variety of cancer cell lines and *in vivo* in animal models [14, 15, 16, 17, 18, 19]. The molecular mechanisms of the action of celastrol have been investigated, and they include modulating apoptosis, proliferation, angiogenesis, invasion, and metastasis [19, 20, 21]. Furthermore, celastrol can sensitize resistant cancer cells to chemotherapy and potentiate radiotherapy [22, 23]. Clinical experience has demonstrated that celastrol possesses great therapeutic potential, and the various pharmacological properties of celastrol may circumvent its toxic drawbacks, thus supporting the increasing number of studies on celastrol designed to further advance drug discovery and development [9]. Despite the large potential of celastrol as an antitumor agent, it exhibits several important limitations for clinical application, including systemic toxicity and adverse effects. However, the development of nanotechnology and synergistic approaches may lead to strategies that can amend the therapeutic potential of celastrol and distribute the drug at target sites.

Elucidating the molecular mechanisms that may ultimately contribute to more effective therapeutic strategies is essential and important. Among the molecular mechanisms dysregulated in cancer, accumulating evidence indicates that mitophagy is associated with cancer progression, metastasis, and chemotherapy resistance [24]. Mitophagy is a selective type of autophagic degradation of mitochondria, and PINK1/Parkin is one of the best‐known mitophagy pathways. Mitophagy has been shown to facilitate tumorigenesis and cell survival in many types of cancers by removing dysfunctional mitochondria [25, 26]. However, excessive mitophagy may cause cell metabolic disorders and eventually lead to cell death. Thus, manipulating mitophagy to improve the efficacy of cancer therapy has received considerable research interest.

In this study, we showed that the combination of celastrol and erastin synergistically induced the cell death of NSCLC cells *in vitro* and *in vivo*. The combination of celastrol and erastin exerted powerful beneficial effects essentially through an increase in ROS levels and the induction of mitochondrial fission and mitophagy. Our data provide the basis for further clinical evaluations of this combination for NSCLC treatment.

## 2 Materials and methods

### 2.1 Antibodies and reagents

The antibodies against p62 (1 : 1000, 5114S), DRP1 (1 : 1000, 8570), phospho‐DRP1 (1 : 1000, 3455), p38 (1 : 1000, 8690), phospho‐p38 (1 : 1000, 4511), PINK1 (1 : 1000, 6949), Parkin (1 : 1000, 4211), OPA1 (1 : 1000, 67589), MFF (1 : 1000, 84580), Mfn1 (1 : 1000, 14739), Mfn2 (1 : 1000, 9482), and cleaved caspase‐3 (1 : 1000, 9664) was purchased from Cell Signaling Technology (Danvers, MA, USA). Antibodies against HSF1‐S326 (1 : 1000, ab76760), FIS1 (1 : 500, ab71498), FTL (1 : 1000, ab47369), FTH (1 : 1000, ab47369), and FPN1 (1 : 1000, ab85370) were purchased from Abcam (Cambridge, MA, USA). The antibody against TOMM20 was purchased from Santa Cruz Biotechnology (Delaware, CA, USA). The antibody against HSF1 (1 : 1000, ABE1044) was obtained from Millipore (Bedford, MA, USA). Antibodies against TFR1 (1 : 1000, 10084‐2‐AP), Lamin A/C (1 : 500, 10298‐1‐AP), Bcl‐2 (1 : 1000, 12789‐1‐AP), Bax (1 : 1000, 50599‐2‐Ig), Nrf2 (1 : 1000, 16396‐1‐AP), NQO1 (1 : 1000, 11451‐1‐AP), GCLC (1 : 1000, 12601‐1‐AP), and β‐actin (1 : 1000, 66009‐1‐Ig) were purchased from ProteinTech (Wuhan, China). Erastin (S7242), celastrol (S1290), PD98059 (S1177), Z‐VAD‐FMK (S7023), Ferrostatin‐1 (S1177), KRIBB11 (S8402), and NAC (S1623) were purchased from Selleck Chemicals (Houston, TX, USA). DFO (D9533) and FAC (RES20400‐A7) were purchased from Sigma‐Aldrich (St. Louis, MO, USA).

### 2.2 Cell culture and treatments

The human NSCLC cell lines HCC827, A540, and H1299 were cultured in RPMI‐1640 medium (Gibco). All these cell lines were obtained from the American Type Culture Collection (ATCC, Manassas, VA, USA). All media consisted of 10% fetal bovine serum and antibiotics. Cells were treated with different concentrations of erastin or/and celastrol for 24 h. For the other cotreatments, the cells were cotreated with erastin and celastrol in the presence of different inhibitors or agents.

### 2.3 Cell Counting Kit‐8 (CCK8) assay

Mitochondrial membrane potential was examined with a CCK‐8 kit (MCE, Shanghai, China) according to the manufacturer’s instructions. Briefly, 10 µL of CCK‐8 solution was added to each well and incubated at 37 °C for 2–3 h. The OD was measured at a wavelength of 450 nm with a microplate reader (BioTek, Winooski, VT, USA).

### 2.4 Propidium iodide (PI) assay

Propidium iodide uptake was measured by flow cytometry to assess cell death. After treatment, cells were stained with 1 μg·mL^−1^ PI (Invitrogen, Eugene, OR, USA) for 30 min at room temperature in the dark. PI uptake was quantified with a FACSCalibur Flow Cytometer (BD Biosciences, San Jose, CA, USA).

### 2.5 Apoptosis assay

Apoptosis was examined by flow cytometry according to the manufacturer’s instructions. In summary, after treatment with erastin and/or celastrol for 24 h, cells were washed three times with PBS and stained with Annexin V‐FITC and PI for 30 min at room temperature in the dark. The percentages of apoptotic cells were examined by flow cytometry (BD Biosciences). Both early (Annexin V‐positive and PI‐negative) and late (Annexin V‐positive and PI‐positive) apoptotic cells were included in the cell death determinations.

### 2.6 Detection of intracellular ROS and lipid ROS

Intracellular levels of ROS and lipid ROS were examined using 2',7'‐dichlorofluorescein diacetate (H2DCF‐DA, Thermo Fisher Scientific, Mountain View, CA, USA) and BODIPY 581/591 C11 probe (Thermo Fisher Scientific), respectively. To measure ROS production, cells were incubated with DCFH‐DA (20 µm) or BODIPY 581/591 C11 (10 µm) for 30 min at room temperature in the dark, washed three times with PBS, and detected by a FACSCalibur Flow Cytometer (BD Biosciences).

### 2.7 Iron assay

Cellular iron concentrations were measured according to the previously described methods [27]. Briefly, after treatment, the cells were incubated with 0.05 mm calcein/acetoxymethyl ester (17783, Sigma) for 20 min at 37 °C under dark conditions. Then, the cells were washed twice with PBS and analyzed with a FACSCalibur Flow Cytometer (BD Biosciences). Calcein was excited at 488 nm, and fluorescence was measured at 525 nm. The difference in the cellular mean fluorescence reflects the relative quantity of cellular iron levels.

### 2.8 Mitochondrial membrane potential

The mitochondrial membrane potential was evaluated with Rhodamine‐123 staining according to the manufacturer’s instructions. After treatment, the cells were stained with Rhodamine‐123 (10 µg·mL^−1^) in the incubator for 30 min and washed with PBS. The intensity of Rhodamine‐123 staining was measured using flow cytometry.

### 2.9 Western blot analysis

Western blot analysis was performed as previously described [28, 29]. The protein concentration was quantified using a BCA protein assay kit. Briefly, 20–40 mg of protein was loaded onto SDS/polyacrylamide gels, and separated proteins were transferred onto polyvinylidene difluoride (PVDF) membranes (Millipore). The membranes were blocked and then incubated with primary antibodies. After washing, the membranes were incubated with an HRP‐conjugated anti‐rabbit or anti‐mouse secondary antibody for 1 h at room temperature.

### 2.10 RNA isolation and quantitative RT‐PCR

Total RNA was extracted from HCC827 cells using a TRIzol‐based method as previously described [28, 29]. The primers used were as follows: HSF1 forward, 5’‐TGAAAAGTGCCTCAGCGTAGCC‐3’, HSF1 reverse, 5’‐TGCTCAGCATGGTCTGCAGGTT‐3’; HSP110 forward, 5’‐GCTCAACAAACCTCACAGTCTCC‐3’, HSP110 reverse, 5’‐AGCTTCTGGAGGCTGGTCAACT‐3’; HSP70 forward, 5’‐ACCTTCGACGTGTCCATCCTGA‐3’, HSP70 reverse, 5’‐TCCTCCACGAAGTGGTTCACCA‐3’; HSP40 forward, 5’‐AGTTCAAGGAGATCGCTGAGGC‐3’, HSP40 reverse, 5’‐GCTGAAAGAGGTACCATTGGCAC‐3’; HSP27 forward, 5’‐CTGACGGTCAAGACCAAGGATG‐3’, HSP27 reverse, 5’‐GTGTATTTCCGCGTGAAGCACC‐3’; and S18 forward, 5’‐GTTCCGACCATAAACGATGCC‐3’, S18 reverse, 5’‐TGGTGGTGCCCTTCCGTCAAT‐3’. All real‐time PCRs were performed in triplicate using samples derived from three independent experiments.

### 2.11 Immunofluorescence assay

Immunofluorescence assays were performed as previously described to examine protein localization and expression [28, 29]. HCC827 cells were washed with PBS, fixed in 4% paraformaldehyde for 10 min, and permeabilized with PBS containing 0.25% Triton for 10 min at room temperature. After washing, the cells were blocked with 5% nonfat milk in PBS for 30 min and then incubated at 4 °C with different antibodies overnight. The cells were then incubated with a secondary fluorescence antibody (Alexa 488 and Alexa 594, Life Technologies, Carlsbad, CA, USA). Immunofluorescence was examined using an Olympus LX70 fluorescence microscope (Olympus, Tokyo, Japan).

### 2.12 Colony formation assay

HCC827 cells or ATG5‐KO HCC827 cells were seeded at 500 per well in 6‐well plates. After attachment for 24 h, the cells were treated with erastin and/or celastrol. After 10–14 days of incubation for colony formation, the cells were fixed with methanol and stained with 0.1% crystal violet. The plates were photographed, and the colonies were counted. The experiment was performed three times for each group.

### 2.13 Subcellular fractionation, co‐immunoprecipitation (co‐IP), and chromatin immunoprecipitation (ChIP) assay

Subcellular fractionation, co‐IP, and ChIP assays were performed as previously described [28, 29].

### 2.14 Lipid peroxidation assay and cellular glutathione (GSH) measurement

Intracellular malondialdehyde (MDA) was examined using a lipid peroxidation assay kit (Nanjing Jiancheng Bioengineering Institute, Nanjing, China) according to the manufacturer’s instructions. The GSH contents were examined using a commercial GSH quantification kit (Nanjing Jiancheng Bioengineering Institute, Nanjing, China) according to the manufacturer’s instructions. All experiments were repeated three times independently.

### 2.15 Detection of mitochondrial DNA copy number

Total DNA was isolated using a kit according to the manufacturer’s protocol (D0063, Beyotime, Haimen, China) to detect the mitochondrial DNA copy number. Real‐time PCR was performed as described previously [30] with the following primers: for mitochondrial ND1, forward, 5′‐ATGGCCAACCTCCTACTCCT‐3′, reverse, 5′‐GCGGTGATGTAGAGGGTGAT‐3′; and for nuclear S18, forward, 5′‐GTTCCGACCATAAACGATGCC‐3′, reverse, 5′‐TGGTGGTGCCCTTCCGTCAAT‐3′. Mitochondrial‐to‐nuclear DNA ratios were quantified using the ΔΔCt method.

### 2.16 Hematologic parameter analysis

Blood samples from mice were collected and placed into 1.5‐mL tubes anticoagulated by EDTA‐2K for the hematologic study after treatment. White blood cells (WBCs), red blood cells (RBCs), lymphocytes (LYMs), and hemoglobin (HGB) were examined using a blood counter (URIT‐5160Vet, China).

### 2.17 CRISPR‐Cas9‐mediated knockout of ATG5

Stable knockout of ATG5 in HCC827 cells was achieved using a CRISPR‐Cas9 system. Lentivirus was produced by transfecting 293T cells with transferable lenti‐CAS‐puro plasmids (Beyotime). HCC827 cells were infected with lentivirus for 24 h, and puromycin‐resistant cells were collected after selection. The cell infection efficiency was confirmed by western blot.

### 2.18 Xenograft assay

Female BALB/c nude mice (BALB/c‐*Foxn1^nu^*, 5–6 weeks old) were purchased from Huafukang (Beijing, China) and fed a standard animal diet and water. All animal protocols were approved and supervised by the Institutional Animal Care and Use Committee of Hebei Normal University (Approval #: 2020LSC10). HCC827 cells were suspended in a 1 : 1 ratio in medium with Matrigel. Nude mice were inoculated with cells in the right legs. The mice were treated with either vehicle, erastin, celastrol, or the combination of erastin and celastrol by intraperitoneal injection once every day. The body weight and tumor volume were measured. The mice were euthanized 21 days after treatment, and the tumors and organs were excised and either formalin‐fixed or flash‐frozen at −20 °C.

### 2.19 Statistical analysis

All data values are represented as the mean ± SD. The comparisons were performed using two‐tailed unpaired Student’s *t*‐tests or ANOVAs (GraphPad Prism 7, San Diego, CA, USA). The results were considered statistically significant at *P < 0.05, **P < 0.01, and ***P < 0.001.

## 3 Results

### 3.1 Combination of erastin and celastrol synergistically induces the death of NSCLC cells

**Fig. 1:**
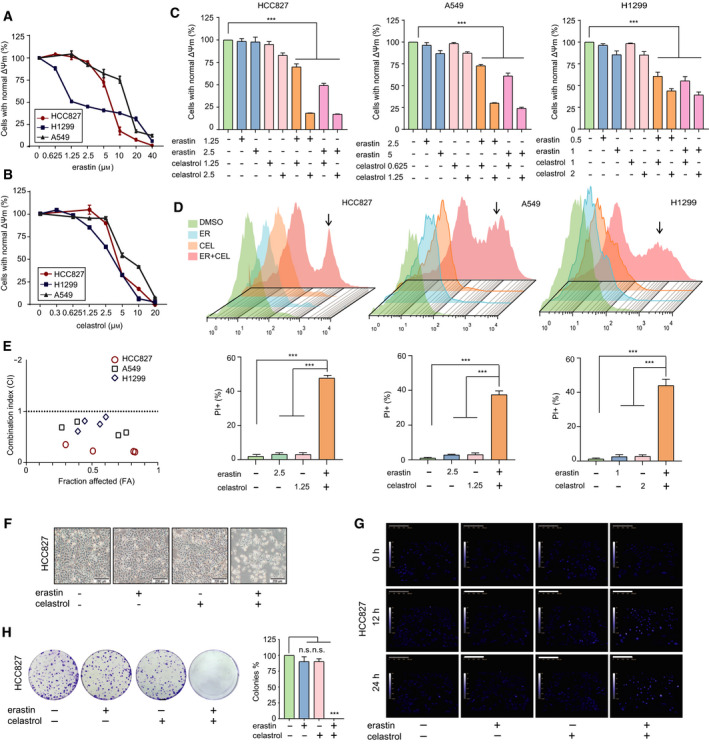
Combination of erastin and celastrol induced NSCLC cell death *in vitro*. (A) HCC827, A549 and H1299 cells were treated with erastin at the indicated concentrations for 24 h, and cell growth was assayed by a CCK‐8 assay. (B) HCC827, A549 and H1299 cells were treated with celastrol at the indicated concentrations for 24 h, and cell growth was assessed by a CCK‐8 assay. (C) HCC827, A549 and H1299 cells were treated with indicated combinations of concentrations of erastin and celastrol for 24 h. Cell viability was measured by a CCK‐8 assay. (D) Cell death was measured by a PI assay using flow cytometry. (E) Combination index (CI) analyses were performed to determine synergy using the Calcusyn software. (F) Representative phase‐contrast microscopic images of HCC827 cells treated with either erastin or celastrol or their combination for 24 h. Most of the cells became smaller in size and round in shape after cotreatment with celastrol and erastin for 24 h. Scale bars, 200 µm. (G) Cell morphology was examined by quantitative holographic phase microscopy. The scale bar indicates the cell height. Scale bars, 200 µm. (H) Representative images of colony formation of HCC827 cells after exposure to either erastin or celastrol, or in combination for 24 h. The mean ± SD is shown, *n* = 3. Statistical significance was determined using two‐tailed unpaired Student’s *t*‐tests. ***P < 0.001. n.s., not significant.

We first treated different NSCLC cell lines with increasing concentrations of erastin or celastrol for 24 h. The results of the CCK‐8 assay showed that either erastin or celastrol decreased cell viability in a dose‐dependent manner in the HCC827, H1299 and A549 cells (Fig. 1A,B). To determine the cytotoxic effect of erastin and celastrol cotreatment, we treated HCC827, A549 and H1299 cells with different combinations of concentrations and examined the cell viability and cell cytotoxicity. Interestingly, we found that low concentrations of the erastin and celastrol cotreatment significantly reduced cell viability and induced cell death; however, either single treatment did not induce a significant cell death effect on these cells (Fig. 1C,D). To confirm this synergism, we used the Calcusyn software to generate combination index (CI) plots. The results showed that all the experimental points had CI values of < 1, indicating that the combination of erastin and celastrol was highly synergistic in NSCLC cells (Fig. 1E and Table 1). Notably, the HCC827 cells were highly sensitive to cotreatment and 2.5 µm of erastin and 1.25 µm of celastrol were selected for further experiments to evaluate the potential synergistic effects of these two agents because this concentration of combination treatment induced half maximal growth inhibition in HCC827 cells (Fig. 1C,D). Moreover, single treatment with erastin or celastrol had no effect on the cell morphology and colony formation in HCC827 cells, while cotreatment significantly altered the cell morphology and prevented colony formation (Fig. 1F–H and Video S1–S4). These results indicate that cotreatment with erastin and celastrol has a strong synergistic anticancer effect on NSCLC cells *in vitro*.

**Table 1:** Combination index (CI) values calculated for erastin and celastrol synergy experiment.

Cell types	Erastin (μm)	Celastrol (μm)	FA	CI
HCC827	1.25	1.25	0.300	0.35022
	1.25	2.5	0.816	0.21593
	2.5	1.25	0.507	0.22554
	2.5	2.5	0.828	0.20731
A549	2.5	0.625	0.271	0.68777
	2.5	1.25	0.700	0.53350
	5.0	0.625	0.388	0.79959
	5.0	1.25	0.760	0.58729
H1299	0.5	1.0	0.393	0.61059
	0.5	2.0	0.561	0.75079
	1	1.0	0.445	0.81331
	1	2.0	0.606	0.89057

### 3.2 Combined regimen of erastin and celastrol did not induce apoptosis or ferroptosis

**Fig. 2:**
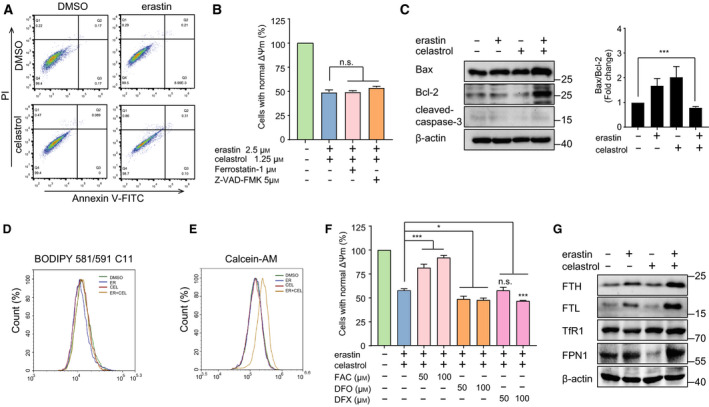
Combination of erastin and celastrol failed to induce apoptosis or ferroptosis. (A) HCC827 cells were treated with erastin and celastrol for 24 h and analyzed using PI/Annexin V‐FITC flow cytometry. (B) HCC827 cells were cotreated with erastin and celastrol in the absence or presence of ferroptosis inhibitor (Fer‐1) or apoptosis inhibitor (Z‐VAD‐FMK) for 24 h. Cell viability was assessed by a CCK‐8 assay. (C) Cleavage of caspase‐3 and expression of Bax and Bcl‐2 were detected by western blotting. (D) Lipid ROS generation was assessed by flow cytometry using C11‐BODIPY. HCC827 cells were treated with either erastin or celastrol or their combination for 24 h. (E) Cellular iron levels were assessed by flow cytometry using Calcein‐AM. HCC827 cells were treated as described in D. (F) HCC827 cells were cotreated with celastrol and erastin in the absence or presence of FAC, DFO, or DFX for 24 h. Cell viability was measured by a CCK‐8 assay (G) Western blot analysis showed that erastin and celastrol cotreatment up‐regulated the expression of TFR1, FPN1, FTH, and FTL in HCC827 cells. The mean ± SD is shown, *n* = 3. Statistical significance was determined using one‐way ANOVA with Tukey’s *post hoc* test. *P < 0.05, ***P < 0.001. n.s., not significant.

To determine whether the synergistic effect of erastin and celastrol was dependent on apoptosis, we examined the percentages of apoptotic cells by flow cytometry. Annexin V/PI‐positive staining indicated that the two drugs, whether alone or in combination, had no significant effect on apoptosis (Fig. 2A). We also treated HCC827 cells with the apoptosis inhibitor Z‐VAD‐FMK and found that Z‐VAD‐FMK did not mitigate erastin‐ and celastrol‐induced cell death (Fig. 2B). Moreover, neither the expression of cleaved caspase‐3 nor the ratio of Bax/Bcl‐2 increased during cotreatment (Fig. 2C). These data suggest that cotreatment with erastin and celastrol did not promote the induction of apoptosis in HCC827 cells.

Because erastin is the classical inducer of ferroptosis, we therefore investigated whether celastrol sensitized the cells to erastin‐induced ferroptosis. Surprisingly, cotreatment with erastin and celastrol did not increase lipid ROS production and lipid peroxidation (Fig. 2D and Fig. S1A), which are two classical features of ferroptosis. The ferroptosis‐specific inhibitor ferrostatin‐1 (Fer‐1) also failed to prevent cell death following erastin and celastrol cotreatment (Fig. 2B). Moreover, the expression of key ferroptosis‐related proteins, including GPX4 and SLC7A11, did not decrease during cotreatment (Fig. S1B). Ferroptosis is dependent on iron accumulation and can be prevented by iron chelators. However, the cellular iron contents significantly decreased following erastin and celastrol treatment (Fig. 2E). In fact, supplementation with the iron chelator DFO or DFX further enhanced the lethality of cotreatment (Fig. 2F). Treatment with ferric ammonium citrate (FAC) greatly inhibited the increase in cell death (Fig. 2F). To further investigate the role of iron, we examined the change in iron‐related protein expression. Ferroportin (FPN1), which is responsible for transferring iron out of cells, was up‐regulated by cotreatment (Fig. 2G). The expression of ferritin (FTH and FTL), which stores cellular iron, was also increased by the erastin and celastrol cotreatment (Fig. 2G). These results imply that although ferroptosis does not occur, the disturbance of iron homeostasis is involved in the cell death induced by celastrol and erastin cotreatment.

### 3.3 Cotreatment with erastin and celastrol induces autophagy in a ROS‐dependent manner

**Fig. 3:**
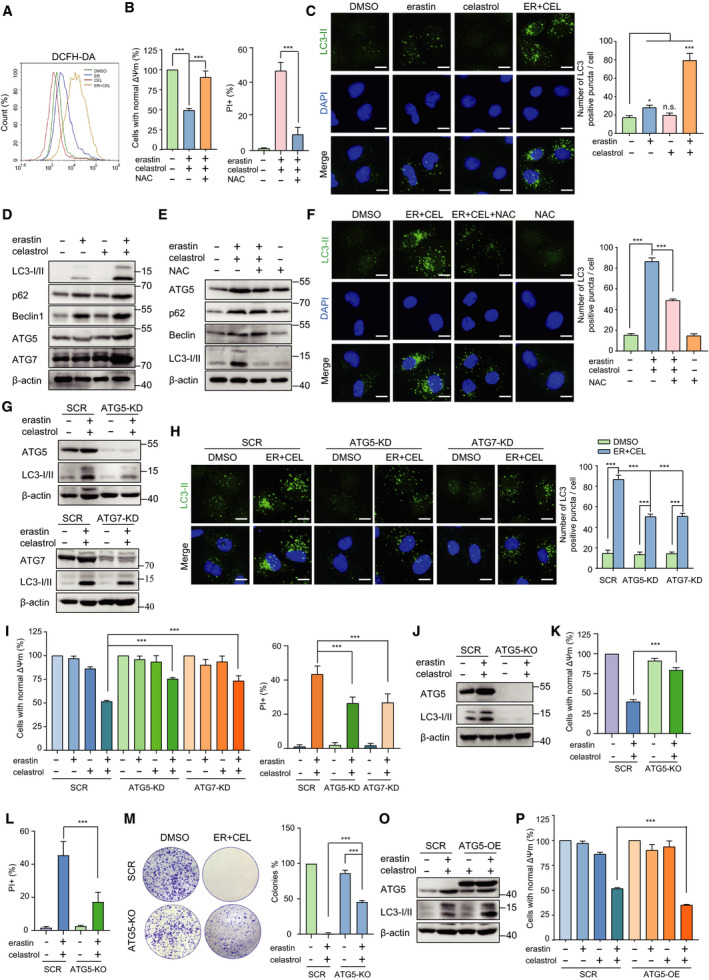
Cotreatment with celastrol and erastin induced ROS‐dependent autophagy. (A) HCC827 cells were cotreated with celastrol and erastin for 24 h. Cells were stained with DCFH‐DA, and the fluorescence intensity was examined by flow cytometry. (B) HCC827 cells were cotreated with erastin and celastrol in the presence or absence of NAC (0.5 mm) for 24 h, and cell viability and cell death were measured by CCK‐8 and PI assays. (C) Representative images of LC3II puncta formation after treatment with either erastin or celastrol or their combination for 24 h. Scale bars, 10 µm. (D) Autophagy‐related proteins, including ATG5, ATG7, p62, Beclin‐1, and LC3, were detected by western blotting after cotreatment with celastrol and erastin for 24 h. (E) HCC827 cells were cotreated with erastin and celastrol in the presence or absence of NAC for 24 h. Autophagy‐related proteins, including ATG5, ATG7, p62, Beclin‐1, and LC3‐I/II, were detected by western blotting. (F) HCC827 cells were treated as described in E. Representative images of LC3‐II puncta formation are presented. Scale bars, 10 µm. (G) Western blotting demonstrated the knockdown of ATG5 or ATG7 prevented up‐regulation of autophagy‐related genes. (H) Representative images of LC3‐II puncta formation in SCR, ATG5‐KD, and ATG7‐KD cells. All the indicated cells were treated with erastin and celastrol for 24 h. Scale bars, 10 µm. (I) CCK‐8 and PI assays showing that ATG5 or ATG7 knockdown mitigated cell death induced by cotreatment with celastrol and erastin. (J) ATG5 knockout in HCC827 cells. (K, L) ATG5 knockout substantially alleviated erastin‐ and celastrol‐induced cell death. (M) Representative images of colony formation of SCR and ATG5‐KO cells after erastin and celastrol cotreatment. (O) Western blotting showing overexpression of ATG5 enhanced LC3‐II up‐regulation. (P) Overexpression of ATG5 potentiated cell death induced by cotreatment with celastrol and erastin. The mean ± SD is shown, *n* = 3. Statistical significance was determined using one‐way or two‐way ANOVA with Tukey’s *post hoc* test. ***P < 0.001.

Although the lipid ROS levels did not change, the intracellular ROS levels were significantly increased by cotreatment with celastrol and erastin (Fig. 3A). To evaluate the role of ROS in the combined treatment, the cells were treated with celastrol and erastin in the presence of NAC, which is a ROS scavenger. Interestingly, NAC treatment completely inhibited celastrol‐ and erastin‐induced cell death, suggesting the critical role of ROS in the cytotoxic effect of celastrol and erastin cotreatment (Fig. 3B). Moreover, the expression of redox‐sensitive transcription factor Nrf2 and its target genes, such as NAD(P)H‐quinone oxidoreductase 1 (NQO1) and glutamate/cysteine ligase (GCLC), was significantly down‐regulated in celastrol‐ and erastin‐treated cells (Fig. S2). An increase in ROS levels stimulates autophagy; therefore, we next investigated whether autophagy was initiated following cotreatment. After cotreatment for 24 h, the numbers of LC3 puncta were significantly increased in HCC827 cells (Fig. 3C). We observed a marked increase in autophagic flux as evidenced by the enhanced conversion of LC3‐I to LC3‐II following erastin and celastrol treatment (Fig. 3D). Moreover, the protein levels of autophagy‐related markers, including Beclin‐1, ATG5, ATG7, and SQSTM1/p62, were significantly up‐regulated in response to celastrol and erastin cotreatment (Fig. 3D). The NAC treatment greatly inhibited the up‐regulated expression of these autophagy‐related genes and the formation of LC3 puncta, indicating that the initiation of autophagy is strongly dependent on ROS (Fig. 3E,F).

To further confirm the autophagy‐dependent cell death, we genetically knocked down the autophagy essential gene ATG5 or ATG7. Consistent with our speculation, knocking down ATG5 or ATG7 significantly inhibited autophagy as indicated by the decreased expression of LC‐3II and reduced autophagic vesicles (Fig. 3G,H). ATG5 and ATG7 knockdown also alleviated the loss of cell viability and increased cell death after 24 h of erastin and celastrol cotreatment (Fig. 3I). In addition, we also generated a stable ATG5 knockout (ATG5‐KO) cell line using the CRISPR‐Cas9 system (Fig. 3J). Knockout of ATG5 significantly alleviated the decreased cell viability and increased cell death after erastin and celastrol cotreatment (Fig. 3K,L). Moreover, knockout of ATG5 also partially rescued colony formation following erastin and celastrol cotreatment (Fig. 3M). Conversely, the overexpression of ATG5 potentiated the process of autophagy and exacerbated erastin‐ and celastrol‐induced cell death (Fig. 3O,P). Taken together, these results strongly indicate that ATG5/ATG7‐mediated autophagy is required for erastin‐ and celastrol‐induced cell death.

### 3.4 Cotreatment with celastrol and erastin disrupts the mitochondrial membrane potential and induces mitochondrial dysfunction

**Fig. 4:**
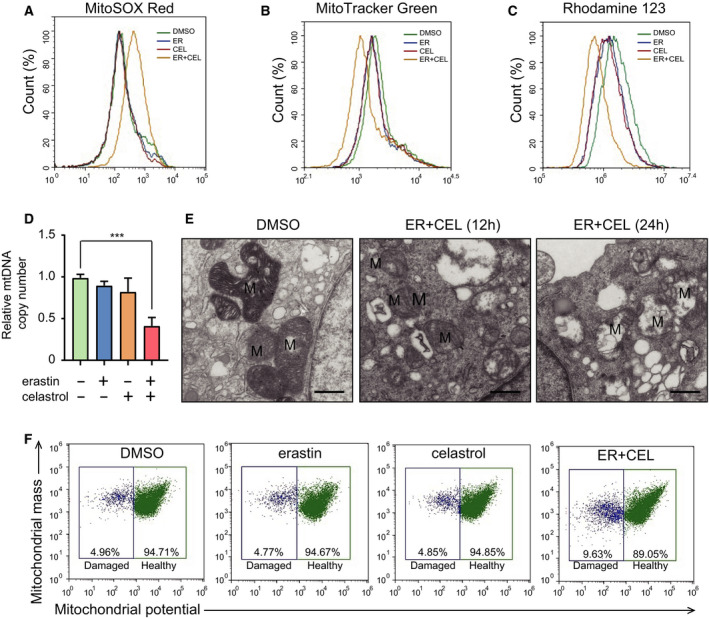
Cotreatment with celastrol and erastin induced mitochondrial dysfunction in HCC827 cells. (A) Mitochondrial ROS generation was assessed using flow cytometry using the MitoSOX™ Red mitochondrial superoxide indicator. (B) Mitochondrial membrane potential was determined after treatment. Cells were stained with a Rhodamine‐123 probe and analyzed by flow cytometry. (C) Mitochondrial mass was assessed by flow cytometry after staining with the MitoTracker Green probe. (D) Mitochondrial copy number was examined by real‐time PCR. (E) TEM was used to indicate the mitochondrial morphology. Intact cristae and normal morphological characteristics were observed in HCC827 cells. Swollen mitochondria with fractured cristae appeared in celastrol‐ and erastin‐treated cells. M, mitochondria. Scale bars, 0.5 µm. (F) Flow cytometry to distinguish populations with normal membrane potential (healthy mitochondria) from those with reduced membrane potential (damaged mitochondria) among HCC827 cell populations. The mean ± SD is shown, *n* = 3. Statistical significance was determined using one‐way ANOVA with Tukey’s *post hoc* test. ***P < 0.001.

Because mitochondria are the major source of ROS and present susceptibility to oxidative stress, we next examined the effect of celastrol and/or erastin on mitochondrial functions. As shown in Fig. 4A, MitoSOX staining showed that erastin and celastrol remarkedly increased the levels of mitochondrial ROS. Although celastrol or erastin alone slightly reduced the mitochondrial membrane potential and mass, their combination greatly augmented the decrease in mitochondrial mass and mitochondrial membrane potential (Fig. 4B,C). The mitochondrial copy number was also markedly decreased in response to erastin and celastrol cotreatment (Fig. 4D). To further identify the damage to mitochondria, TEM was used to observe the ultrastructural changes in HCC827 cells. The mitochondria presented intact cristae and normal morphological characteristics in HCC827 cells but appeared swollen and lacked ruptured cristae in cells cotreated with celastrol and erastin (Fig. 4E). Consistent with these results, the abundance of damaged mitochondria with low potential increased following the celastrol and erastin cotreatment (Fig. 4F). These data indicate that celastrol and erastin cotreatment could induce the depolarization of the mitochondrial membrane potential and increase mtROS levels, thus leading to the accumulation of damaged mitochondria.

### 3.5 Cotreatment with celastrol and erastin induces mitophagy

**Fig. 5:**
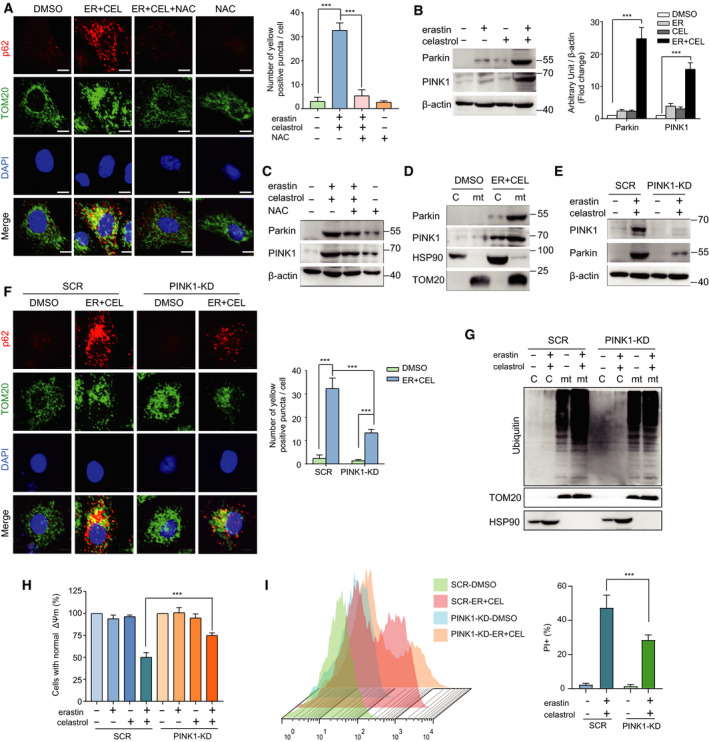
Cotreatment with celastrol and erastin induced PINK1/Parkin‐dependent mitophagy. (A) Colocalization of p62 and TOM20 was visualized under confocal microscopy. Representative fluorescent images of cells immunostained with p62 and TOM20 after erastin and celastrol cotreatment for 24 h are shown. The yellow dots in the merge panel indicated the activation of mitophagy. Scale bars, 10 µm. (B) Western blotting was used to measure the protein levels of PINK1 and Parkin in HCC827 cells treated with celastrol and erastin for 24 h. (C) HCC827 cells were cotreated with erastin and celastrol in the presence or absence of NAC for 24 h. The expression levels of PINK1 and Parkin were detected by western blotting. (D) Western blotting analysis of PINK1 and Parkin in the cytosolic and mitochondrial fractions of HCC827 cells treated with erastin and celastrol for 24 h. (E) Western blotting analysis of the effect of PINK1 knockdown on the expression of Parkin. (F) Colocalization of p62 and TOM20 was reduced in PINK1‐KD cells. Representative fluorescent images of cells immunostained with p62 and TOM20 after cotreatment with celastrol and erastin for 24 h are shown. Scale bars, 10 µm. (G) Ubiquitination levels of protein in the cytosolic and mitochondrial fractions in SCR and PINK1‐KD cells were determined by western blotting. (H, I) Cell viability and cell death were measured by CCK‐8 and PI assays. The mean ± SD is shown, *n* = 3. Statistical significance was determined using two‐way ANOVA with Tukey’s *post hoc* test. ***P < 0.001.

The disruption of mitochondria is an important effect of celastrol and erastin cotreatment, and the clearance of damaged mitochondria is mediated by mitophagy. To determine whether mitophagy is involved in the cell death induced by erastin and celastrol cotreatment, we examined the colocalization of endogenous p62 and TOM20, which is a mitochondrial marker. The number of p62 puncta colocalizing with TOM20 greatly increased following cotreatment, indicating that mitophagy was stimulated by erastin and celastrol cotreatment (Fig. 5A). Notably, the colocalization was significantly decreased in the presence of NAC, suggesting that mitophagy was also dependent on ROS (Fig. 5A). Recent studies have revealed that the PINK1/Parkin signaling pathway is a key pathway in mitophagy regulation. As shown in Fig. 5B, erastin and celastrol treatment significantly increased the expression of PINK1 and Parkin. Moreover, NAC treatment markedly suppressed the increase in PINK1 and Parkin expression (Fig. 5C). To examine the impact of the erastin and celastrol treatment on PINK1/Parkin translocation between the cytosol and mitochondria, we isolated the cytosolic and mitochondrial fractions from HCC827 cells. The translocation of PINK1 and Parkin into the mitochondria was greatly enhanced in the mitochondrial fraction following erastin and celastrol cotreatment (Fig. 5D). Parkin is an E3 ubiquitin ligase that ubiquitinates mitochondria to promote their degradation by mitophagy. Indeed, after cotreatment with erastin and celastrol for 24 h, the ubiquitinated protein significantly accumulated in the mitochondrial fraction (Fig. 5G).

To further clarify the role of mitophagy in cytotoxicity, we knocked down the expression of PINK1. Silencing PINK1 partially prevented the cotreatment‐stimulated increase in Parkin expression (Fig. 5E). In addition, PINK1 knockdown blocked the colocalization of p62 with TOM20, indicating mitophagy inhibition (Fig. 5F). The ubiquitinated protein level in mitochondria was also greatly reduced in PINK1 knockdown cells (Fig. 5G). More importantly, PINK1 knockdown inhibited cell death (Fig. 5H,I). Therefore, erastin and celastrol cotreatment induces PINK1/Parkin‐dependent mitophagy in HCC827 cells, which contributes to cytotoxicity.

### 3.6 Cotreatment with erastin and celastrol induces mitochondrial fission

**Fig. 6:**
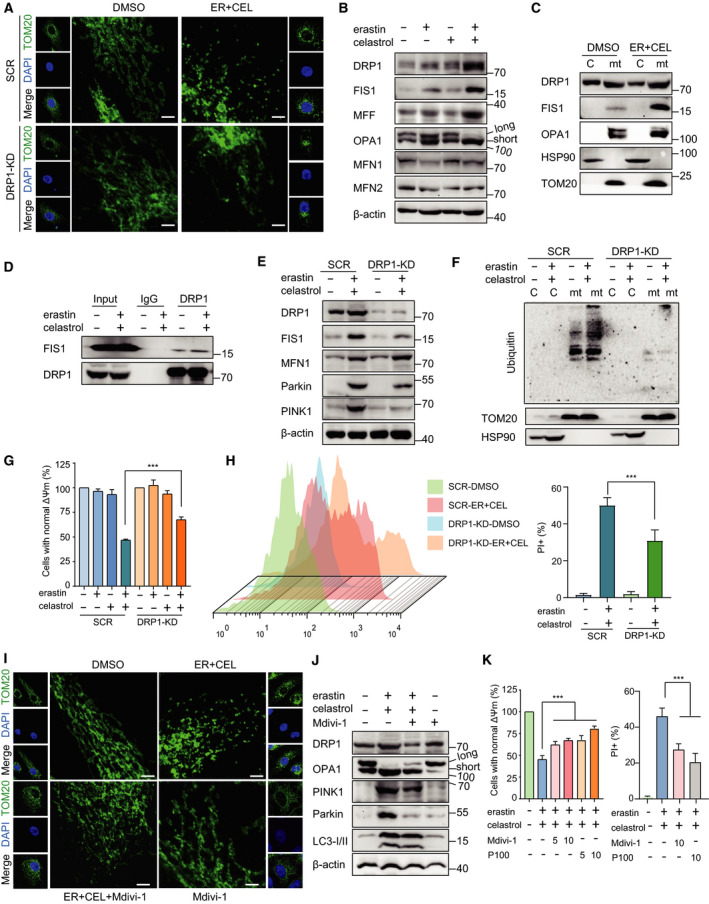
Cotreatment with celastrol and erastin induced DRP1‐mediated mitochondrial fission. (A) Representative fluorescence photographs of mitochondria following erastin and celastrol cotreatment for 24 h. Mitochondrial morphology was analyzed by TOM20 staining. Scale bars, 10 µm. (B) Western blotting analysis of mitochondrial fission proteins (DRP1, OPA1, FIS1, and MFF) and mitochondrial fusion proteins (MFN1 and MFN2) in HCC827 cells after cotreatment with celastrol and erastin for 24 h. (C) The cytosolic and mitochondrial proteins were separately extracted. The expression levels of DRP1, FIS1, and OPA1 were evaluated by western blotting. TOM20 and HSP90 were used as the loading controls for mitochondria and cytosol, respectively. (D) Cotreatment enhanced the interaction between DRP1 and FIS1. HCC827 cells were treated as described in A. (E) SCR and DRP1‐KD cells were subjected to subcellular fractionation for the isolation of cytosol and mitochondria for western blotting. (F) Ubiquitination levels of protein in the cytosolic and mitochondrial fractions in SCR and DRP1‐KD cells were determined by western blotting analysis. (G, H) Knockdown of DRP1‐attenuated cell death induced by erastin and celastrol cotreatment. Cell viability and cell death were measured by CCK‐8 and PI assays (I) HCC827 cells were cotreated with erastin and celastrol in the presence or absence of Mdivi‐1 (20 µm) for 24 h. The mitochondrial morphology was analyzed by TOM20 staining. Scale bars, 10 µm. (J) HCC827 cells were treated as described in H, and the expression of mitochondrial fission proteins (DRP1 and OPA1) and mitophagy proteins (PINK1 and Parkin) was examined by western blotting. (K) Cell viability and cell death were measured by CCK‐8 and PI assays. The mean ± SD is shown, *n* = 3. Statistical significance was determined using one‐way or two‐way ANOVA with Tukey’s *post hoc* test. ***P < 0.001.

Increasing evidence suggests that mitochondrial fission promotes the induction of mitophagy; therefore, we further examined the mitochondrial dynamics. The mitochondria in HCC827 cells were long and tubular in the absence of treatment (Fig. 6A). Erastin and celastrol cotreatment significantly increased the number of punctate and short mitochondria and reduced the number of tubular mitochondria (Fig. 6A). To explore the mechanism by which erastin and celastrol cotreatment changes the mitochondrial morphology, the expression of proteins that mediate mitochondrial fission and fusion was investigated. Although the expression of the mitochondrial fusion proteins mitofusin 1 (MFN1) and MFN2 was slightly altered, the expression of mitochondrial fission proteins, including dynamin‐related protein 1 (DRP1), mitochondrial fission 1 protein (FIS1), and mitochondrial fission factor (MFF), was dramatically enhanced in erastin‐ and celastrol‐treated cells (Fig. 6B). We also observed a reduction in the long optic atrophy 1 (L‐OPA1) isoform and the accumulation of the short isoform of OPA1 (S‐OPA1), suggesting the promotion of mitochondrial fission (Fig. 6B). Moreover, the translocation of DRP1, FIS1, and OPA1 into the mitochondria was greatly enhanced in the mitochondrial fraction (Fig. 6C). In response to metabolic cues, DRP1 is recruited to mitochondria and interacts with FIS1 to form foci for fission events. Erastin and celastrol cotreatment enhanced the interaction between DRP1 and FIS1 (Fig. 6D).

To dissect the role of mitochondrial fission in erastin‐ and celastrol‐induced mitophagy, we investigated the effects of the shRNA‐mediated knockdown of DRP1. The knockdown of DRP1 significantly inhibited the mitochondrial fission induced by erastin and celastrol cotreatment, which was evidenced by the restoration of the elongated rod‐shaped structure of mitochondria (Fig. 6A). Furthermore, DRP1 knockdown inhibited the increase in PINK1 and Parkin expression and the ubiquitinated protein level in mitochondria following erastin and celastrol cotreatment (Fig. 6E,F). More importantly, the silencing of DRP1 significantly blocked erastin‐ and celastrol‐induced cell death, indicating that DRP1 knockdown attenuated mitochondrial fragmentation and cell death (Fig. 6G,H). To further explore the function of DRP1 in mitochondrial fission and cell death, we used Mdivi‐1 and P110 to selectively inhibit DRP1 but no other proteins that regulate mitochondrial dynamics. We found that Mdivi‐1 not only reduced the mitochondrial fission process but also greatly mitigated the erastin‐ and celastrol‐induced stimulation of mitophagy (Fig. 6I,J). Both Mdivi‐1 and P110 significantly inhibited the altered cell viability and cell death (Fig. 6K). Therefore, these results indicate that erastin‐ and celastrol‐induced mitophagy is mediated by the upstream DRP1‐regulated mitochondrial fission process.

### 3.7 p38 interacts with and phosphorylates DRP1 following cotreatment with celastrol and erastin

**Fig. 7:**
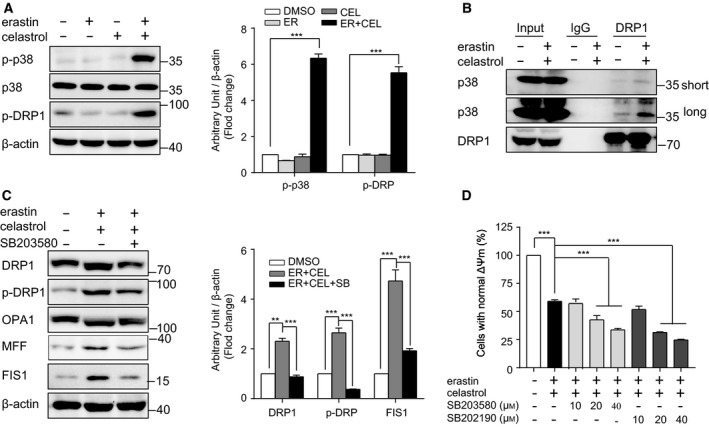
Activation of p38 promotes mitochondrial fission induced by cotreatment with celastrol and erastin. (A) Western blotting showed that phosphorylation of p38 and DRP1 was increased by cotreatment with celastrol and erastin. (B) Cotreatment with celastrol and erastin enhanced the interaction between DRP1 and p38. Complexes coimmunoprecipitated using antiserum against DRP1 were blotted with p38 or DRP1 antibodies. (C) Inhibition of p38 alleviated mitochondrial fission. The expression of DRP1, OPA1, and FIS1 was examined by western blotting. (D) CCK‐8 assay demonstrated that inhibition of p38 by SB203580 or SB202190 further exacerbated the cell death induced by cotreatment with celastrol and erastin. The mean ± SD is shown, *n* = 3. Statistical significance was determined using one‐way ANOVA with Tukey’s *post hoc* test. ***P < 0.001.

p38 has been reported to regulate DRP1 activity by promoting the phosphorylation of Ser 616 to induce mitochondrial fission [31]. We found increased DRP1 phosphorylation at Ser 616 following erastin and celastrol cotreatment (Fig. 7A). Moreover, the phosphorylation of p38 was enhanced by cotreatment, indicating the activation of p38 (Fig. 7A). The interaction between p38 and DRP1 was greatly increased in erastin‐ and celastrol‐treated cells (Fig. 7B). Furthermore, we pharmacologically inhibited p38 activity using the specific inhibitor SB203580. As shown in Fig. 7C, SB203580 blocked the phosphorylation of DRP1 at Ser 616 and inhibited the increased expression of FIS1 and MFF. However, coincubation with either SB203580 or SB202930 further enhanced the cell death caused by erastin and celastrol cotreatment (Fig. 7D). Although these results were unexpected, they suggest that DRP1 is not the only substrate for p38 and that p38 may regulate other targets or pathways to promote cell survival during erastin and celastrol treatment.

### 3.8 Inhibition of HSF1 aggravated the celastrol and erastin combination‐induced cell death

**Fig. 8:**
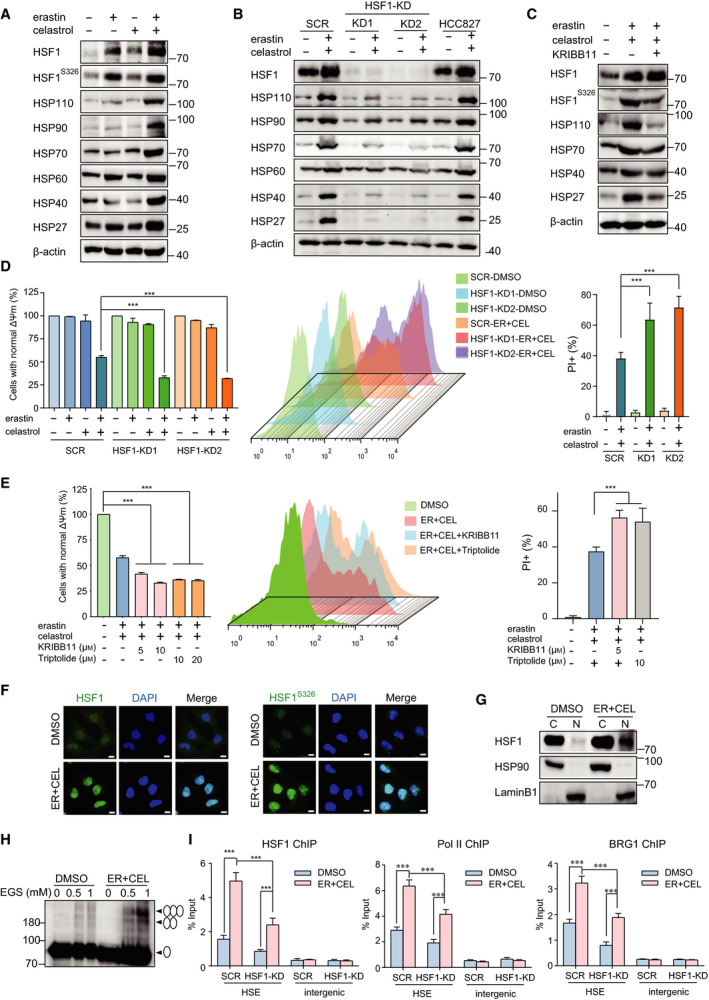
HSF1 activation and HSP expression are associated with the resistance to cotreatment with celastrol and erastin. (A) Protein levels of HSP and HSF1 in cells exposed to erastin or/and celastrol for 24 h. (B) Western blotting showed that knockdown of HSF1 with shRNA inhibited up‐regulation of HSPs. (C) Inhibition of HSF1 by KRIBB11 reduced the up‐regulation of HSP expressions. The phosphorylation of HSF1 and protein levels of HSP was investigated by western blotting. (D) Knockdown of HSF1 promoted cell death induced by cotreatment with celastrol and erastin in HCC827 cells. Cell death was measured by CCK‐8 and PI assays (E) CCK‐8 and PI assays demonstrated that inhibition of HSF1 by KRIBB11 or triptolide exacerbated the cell death induced by cotreatment with celastrol and erastin. (F) Erastin and celastrol cotreatment significantly increased HSF1 phosphorylation at S326 and HSF1 nuclear translocation. Immunofluorescence staining using antibodies against p‐HSF1 S326 and HSF1 was observed using a confocal microscope. Scale bars, 10 µm. (G) Nuclear translocation of HSF1 is enhanced by cotreatment with celastrol and erastin. HCC827 cells were fractionated into cytoplasmic and nuclear proteins. Lamin B1 and HSP90 were used as nuclear and cytoplasmic markers, respectively. (H) Cross‐linking experiment showed that cotreatment with celastrol and erastin promoted HSF1 trimer formation. (I) HSF1, BRG1, and Pol II recruitment to the HSP70 promoter was enhanced by cotreatment with celastrol and erastin. SCR and HSF1‐KD HCC827 cells were treated as described in A, and ChIP‐qPCR analyses were performed using HSF1, BRG1 and Pol II antibodies. The mean ± SD is shown, *n* = 3. Statistical significance was determined using one‐way or two‐way ANOVA with Tukey’s *post hoc* test. ***P < 0.001.

The disturbance of protein homeostasis is another cause of cell death [32]. Heat shock proteins (HSPs) are constitutively expressed and/or stress‐induced molecules, and they show not only a chaperone activity in nascent proteins but also a remedial role following cell stress [33]. HSP guarantees cell tolerance against a variety of stressors, such as heat shock, oxidative stress, infection, and inflammation. Next, we investigated whether protein homeostasis was lost following erastin and celastrol cotreatment. Cotreatment with erastin and celastrol significantly increased the mRNA levels of HSP110, HSP70, HSP40, and HSP27 in a time‐dependent manner (Fig. S3A). Moreover, the cotreatment obviously enhanced the protein levels of these HSPs (Fig. 8A). Heat shock factor 1 (HSF1) is the master orchestrator of HSP expression; therefore, we investigated HSF1 expression following treatment. HSF1 protein levels greatly increased when the cells were cotreated with erastin and celastrol (Fig. 8A). To investigate the function of HSF1 in regulating HSP expression, HSF1 was silenced by two different shRNAs. HSF1 knockdown not only reduced constitutive HSP expression but also significantly inhibited HSP induction following erastin and celastrol cotreatment (Fig. 8B). Moreover, treatment with KRIBB11, an HSF1 inhibitor, also markedly prevented HSP expression (Fig. 8C). HSF1 knockdown or inhibition by KRIBB11 or triptolide further reduced cell viability and exacerbated cell death (Fig. 8D,E). In contrast, HSF1 overexpression or activation greatly alleviated cell death (Fig. S3C,D). These results suggest that erastin and celastrol cotreatment promotes HSP induction in an HSF1‐dependent manner.

The elevated protein level of HSF1 indicated that HSF1 might be activated by erastin and celastrol cotreatment. The phosphorylation of HSF1 at S326, a dominant protein modification that is critical for HSF1 activation, was significantly enhanced following cotreatment (Fig. 8A,F). The phosphorylation of HSF1 is associated with the trimer formation and nuclear translocation of HSF1, which are needed for HSF1 activation [34]. Immunofluorescence and subcellular fractionation analyses suggested that HSF1 translocated and accumulated in the nucleus from the cytoplasm following erastin and celastrol treatment (Fig. 8F,G). Cotreatment also enhanced HSF1 trimer formation (Fig. 8H). To further explore the in‐depth mechanism by which cotreatment promoted HSF1 activation, we performed ChIP assays. Cotreatment significantly enhanced the recruitment of HSF1 to the HSP70 promoter (Fig. 8I). This recruitment of HSF1 was greatly impaired by HSF1 knockdown (Fig. 8I). Previous studies have shown that the promoter‐proximal pausing of RNA polymerase II (Pol II) and recruitment of transcriptional coactivators enable synchronized gene expression in a large number of HSP genes upon the reception of gene activation signals [32]. As expected, the occupancy of Pol II on the HSP70 promoter increased following erastin and celastrol treatment (Fig. 8I). The recruitment of another transcriptional co‐activator, BRG1, a subunit of the chromatin‐remodeling complex, was also significantly enhanced. However, HSF1 knockdown greatly impeded the recruitment of these factors to the HSP70 promoter, indicating that HSF1 facilitates the recruitment of Pol II and BRG1 to HSP promoters in response to erastin and celastrol cotreatment (Fig. 8I). In summary, these results demonstrate that cotreatment with erastin and celastrol promotes phosphorylation at S326, nuclear translocation of HSF1, and recruitment of HSF1 to HSP promoters to amplify HSP induction.

### 3.9 Cotreatment with celastrol and erastin prevents the proliferation of HCC827 cells *in vivo*

**Fig. 9:**
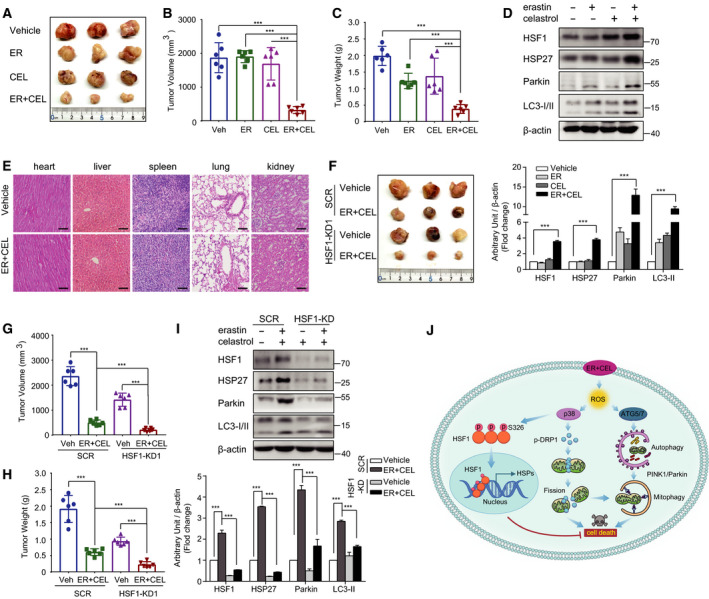
Antitumor activity of erastin and celastrol cotreatment on xenograft models of NSCLC. (A) Nude mice were injected subcutaneously with indicated HCC827 cells. Images of isolated tumors from nude mice treated with single‐agent erastin (5 mg·kg^−1^), celastrol (1 mg·kg^−1^), and their combination. Tumor volumes (B) and weights (C) of HCC827 xenografts in each treatment group are shown. (D) Tumor lysates were subjected to western blotting for HSF1, HSP27, LC3, and Parkin. (E) H&E staining tissue images obtained from major organs of erastin or/and celastrol‐treated mice for the *in vivo* toxicity evaluation. Scale bar, 100 µm. (F) HSF1 knockdown HCC827 cells were more sensitive to erastin and celastrol cotreatment *in vivo*. Representative images of subcutaneous tumors in mice inoculated with stable HSF1 knockdown HCC827 cells compared with the control group. Tumor volumes (G) and weights (H) of subcutaneous tumors in mice inoculated with stable HSF1 knockdown or SCR HCC827 cells. (I) Western blotting was performed to examine HSF1, HSP27, LC3, and Parkin protein expression in subcutaneous tumors. (J) Proposed mechanism of celastrol‐ and erastin‐induced cell death in NSCLC. The mean ± SD is shown, *n* = 6. Statistical significance was determined using one‐way or two‐way ANOVA with Tukey’s *post hoc* test. ***P < 0.001.

Given that the celastrol and erastin cotreatment effectively induced NSCLC cell death *in vitro*, we next investigated their combined effect *in vivo* using a xenograft mouse model. We inoculated nude mice with HCC827 cells and treated mice with celastrol (1 mg·kg^−1^) and/or erastin (5 mg·kg^−1^) or vehicle for 3 weeks. Compared with celastrol or erastin treatment alone, HCC827 xenograft model mice treated with the combination exhibited increased antitumor activity (Fig. 9A–C). The body weight did not significantly change among the vehicle control, celastrol treatment alone, erastin treatment alone, and celastrol and erastin combination, indicating that the combination of celastrol and erastin is well tolerated and efficiently prevents tumor growth *in vivo* (Fig. S4A,B). Moreover, the hematopoietic indexes were evaluated. The counts of WBCs and RBCs in the four different groups did not exhibit significant differences (Fig. S4C,D). In addition, significant differences in LYM and HGB were not observed in all exposed groups compared with the vehicle group (Fig. S4E,F). Similar to the *in vitro* results, celastrol and erastin cotreatment also increased the expression of LC3‐II, Parkin, HSF1, and HSP27 in tumor tissues (Fig. 9D). Moreover, significant signs of toxicity were not observed in the hearts, livers, spleens, lungs, and kidneys during the celastrol and/or erastin treatment (Fig. 9E). We also investigated the role of HSF1 *in vivo* and found that the tumor volumes and weights were further reduced in HSF1‐knockdown mice compared with the scrambled control mice after cotreatment with erastin and celastrol (Fig. 9F–H). The knockdown efficiency was confirmed by the reduced protein levels of HSF1 and HSP27 in tumor tissues (Fig. 9I). Moreover, HSF1 silencing also attenuated the up‐regulation of Parkin and LC3‐II (Fig. 9I). These results demonstrate that celastrol and erastin cotreatment exerts anticancer effects on NSCLC xenografts and that the inhibition of HSF1 further potentiated this synergistic therapeutic effect.

## 4 Discussion

Celastrol, a pentacyclic triterpenoid, exerts excellent anticancer activity against a broad spectrum of cancers by targeting various molecules and pathways that are important for cancer development and progression [16]. Moreover, celastrol can potentiate the anticancer effects of chemotherapeutic drugs used in the clinic [22, 23]. Despite its remarkable pharmacological effects, the clinical application of celastrol is restricted due to its severe adverse effects and poor bioavailability. Combination therapy is a promising strategy to overcome the compensatory mechanisms and unwanted off‐target effects that limit the utility of many potential drugs, and it can disrupt cancer cell homeostasis and/or metabolism through various simultaneous targets or pathways to improve the therapeutic treatment efficacy, decrease the drug dosage, reduce adverse effects, and inhibit or delay the development of acquired resistance [35, 36]. In the present study, we found that the combination of celastrol and erastin could synergistically inhibit NSCLC cell growth *in vitro* and *in vivo*. The increase in ROS, mitochondrial dysfunction, ATG5/ATG7‐dependent autophagy, mitochondrial fission, and Parkin/PINK1‐dependent mitophagy contributed to the anticancer activity of the combination of celastrol and erastin (Fig. 9J). More importantly, when used in combination, the concentration of celastrol is low, which reduces the side effects of celastrol. No significant changes in body weight and hematologic parameters were observed in the mice, and no significant damage was found in the major organs (Fig. 9 and Fig. S3). Our data provide a conceptual framework for the development of a novel strategy for combined treatment utilizing erastin and celastrol.

Redox homeostasis is pivotal for cell metabolism and cell survival, while ROS, which are produced under physiological and pathological conditions, present dichotomous roles in the cell [37]. Low levels of ROS act as secondary messages to regulate cellular signaling pathways, such as cell growth, differentiation, and survival. However, when ROS levels are excessively high, cell death is induced through oxidative damage to DNA, proteins, lipids, and cell structures [38]. The exacerbation of oxidative stress by enhancing ROS generation is a promising potential anticancer strategy and represents a mechanism of action of various current anticancer approaches that have been used in clinical practice [38, 39]. Because many cancer cells have developed resistance to apoptosis, the activation of this cell death pathway by ROS may not be sufficient to kill all cancer cells. The possibility of using molecules that exacerbate ROS generation and elicit other types of cell death is a promising scenario for ROS‐based cancer therapies. Therefore, the number of natural and synthetic molecules with these interesting properties is continually increasing. In the present study, the chemosensitizing effect of the combination of celastrol and erastin is hypothesized to be associated with oxidative stress as indicated by the attenuation of ROS generation and cytotoxicity upon NAC treatment (Fig. 3B). Although ROS are not essential for cell death, these molecules are indispensable for autophagy and mitophagy induction. These data imply that the chemosensitizing effect is causally associated with ROS accumulation and redox stress.

Iron is an essential metal in living organisms that it is necessary for cellular metabolism, growth, and proliferation. Increasing evidence indicates that iron homeostasis is deregulated in cancer [40, 41]. Several epidemiological reports have suggested a positive relationship between the risk of cancer and high body iron contents in the general population [42]. Given the high iron needs of cancer cells to sustain cell proliferation, changes in iron trafficking in cancer cells result in iron acquisition or iron addiction. Thus, iron not only contributes to oncogenesis but also promotes the rapid growth rate of cancer cells. Considering the role of iron in cancer, the manipulation of iron levels could be an effective strategy for both cancer prevention and treatment. Iron chelators, such as DFO, DFX, and thiosemicarbazone Dp44m, have been used to treat several types of cancers [43, 44]. In the present study, we found that erastin and celastrol cotreatment significantly reduced cellular iron levels and that supplementation with FAC effectively reversed cell death (Fig. 2). These results are consistent with the strong up‐regulation of FPN1, FTH, and FTL, indicating that decreasing cellular iron is one of the mechanisms through which erastin and celastrol cotreatment kills cancer cells.

Ferroptosis is a new form of programmed cell death characterized by the accumulation of iron and the production of lipid hydroperoxides at lethal levels [6]. Erastin has been regarded as a new strategy for developing ferroptosis‐based therapeutic agents for highly effective therapies against many cancers [5, 7]. The discovery that erastin potentiates the anticancer activity of celastrol in a ferroptosis‐independent manner is somewhat surprising (Fig. 2) because erastin is a classical inducer that selectively induces ferroptosis. Although erastin has been considered a potent anticancer agent, its ability to increase the cytotoxic effect in combination with celastrol was not anticipated and could not be reasonably explained by its key mechanism of action. Indeed, cellular iron and lipid ROS did not increase following celastrol and erastin cotreatment (Fig. 2). The ferroptosis‐specific inhibitor Fer‐1 also failed to rescue cell viability, indicating that ferroptosis likely does not have a significant role in cellular sensitivity to celastrol and erastin cotreatment.

HSF1 has been identified as a promising and effective drug target because it regulates multiple facets of malignancy, including proliferation, migration, invasion, and metastasis [33, 34, 45]. As the master controller of the heat shock response (HSR) to proteotoxic stresses, HSF1 is crucial in cancers because it enables cancerous cells to copy oncogenic stress or drastic conditions by inducing the expression of cytoprotective chaperone proteins, such as HSP70, HSP40, and HSP27 [33, 34]. HSF1 is also known to induce multidrug resistance genes against both cisplatin and carboplatin treatment. Increased levels of HSF1 are generally associated with poor prognosis. Thus, HSF1 is considered a diagnostic and prognostic biomarker, and it is currently being targeted to develop new cancer therapies. Silencing HSF1 by RNA interference or inhibiting HSF1 by chemical inhibitors sensitizes cancer cells to chemotherapeutic reagents or hyperthermia therapy [28, 46]. A previous study showed that celastrol could induce HSR by promoting HSF1 phosphorylation, increasing DNA binding activity, and up‐regulating HSP70 expression [47]. Moreover, suboptimal concentrations of celastrol and heat shock temperatures caused significant synergistic effects, suggesting that celastrol could lower the threshold for HSR [47]. In addition, erastin has been reported to stimulate HSF1‐dependent HSPB1 expression in cancer cells to confer protection against ferroptosis [48]. Here, we found that low concentrations of erastin and celastrol strongly activated HSF1 and induced HSP expression in an HSF1‐dependent manner to protect cells from proteotoxic stress (Fig. 8A,B). The knockdown or inhibition of HSF1 significantly enhanced cell death through, at least, the suppression HSP induction during erastin and celastrol cotreatment (Fig. 8A,E). Our results suggest that HSF1 activation can attenuate the sensitivity of cancer cells to chemotherapy drugs and that the inhibition of HSF1 may be a novel target for developing a sensitization strategy to NSCLC chemotherapy in the clinic.

## 5 Conclusions

In summary, we found that cotreatment with low concentrations of erastin and celastrol markedly induced cell death through the activation of the ROS–mitochondrial fission–mitophagy signaling pathway, thus providing new insight into the application of erastin and celastrol cotreatment for NSCLC. Additional studies should focus on a potential clinical trial to test the efficacy of erastin and celastrol cotreatment in NSCLC patients.

## Conflict of interest

The authors declare no conflict of interest.

## Author contributions

KT and YF conceptualized the design. ML, DL, BH, YM, FC, TL, ZS, YH, and LH performed formal analysis. KT, YC, PC, and YF performed investigation. AN and KT wrote, reviewed, and edited the manuscript. KT and YF performed funding acquisition. All authors have read and agreed to the published version of the manuscript.

## Supporting information


**Fig. S1.** Combination of erastin and celastrol failed to induce ferroptosis. (A) Measurement of cellular MDA in HCC827 cells exposed to erastin and/or celastrol for 24 h. (B) Western blotting analysis of GPX4 and SLC7A11 expression in HCC827 cells. The mean ± SD is shown, n=3. Statistical significance was determined using one‐way ANOVA with Tukey’s post hoc test. ***p<0.001. n.s., not significant.

**Fig. S2.** Nrf2, NQO1 and GCLC protein levels in cells exposed to erastin or/and celastrol for 24 h. The mean ± SD is shown, n=3. Statistical significance was determined using two‐way ANOVA with Tukey’s post hoc test. ***p<0.001.

**Fig. S3.** Overexpression or activation of HSF1 inhibited HSP induction and cell death induced by cotreatment with celastrol and erastin. (A) Levels of HSP110, HSP70, HSP40 and HSP27 mRNA in HCC827 cells were examined by RT‐qPCR. HCC827 cells were treated with either erastin or celastrol alone or their combination for the indicated amount of time. (B) HSF1 overexpression prevented cell death induced by cotreatment with celastrol and erastin in HCC827 cells. Cell viability was examined by CCK‐8 assays. (C) CCK‐8 assay demonstrated that activation of HSF1 by paeoniflorin or resveratrol inhibited the cell death induced by cotreatment with celastrol and erastin. The mean ± SD is shown, n=3. Statistical significance was determined using one‐way or two‐way ANOVA with Tukey’s post hoc test. **p<0.01, ***p<0.001.

**Fig. S4.** Changes in body weight and hematologic parameters in mice during treatment. (A, B) Time course (days) of body weight after treatment. (C‐F) Changes of hematologic parameters in mice after treatment with erastin and/or celastrol. Blood samples were collected and the hematologic parameters, including WBC, RBC, LYM and HGB, were examined. The mean ± SD is shown, n=6. Statistical significance was determined using one‐way ANOVA with Tukey’s post hoc test. n.s., not significant.

**Video S1.** Changes in cell morphology in HCC827 cells.

**Video S2.** Changes in cell morphology in erastin‐treated HCC827 cells.

**Video S3.** Changes in cell morphology in celastrol‐treated HCC827 cells.

**Video S4.** Changes in cell morphology in HCC827 cells treated with erastin and celastrol.

## Data Availability

The authors declare that all data supporting the findings of this study are available within the article and its additional files, or through contact with the corresponding author upon reasonable request.
