# Supplementary figures and images for: Ferroptosis inducer erastin sensitizes NSCLC cells to celastrol through activation of the ROS–mitochondrial fission–mitophagy axis
Source: Mol Oncol. 2021 Mar 17;15(8):2084–105. doi: 10.1002/1878-0261.12936 (PMC8334255; doi:10.1002/1878-0261.12936)

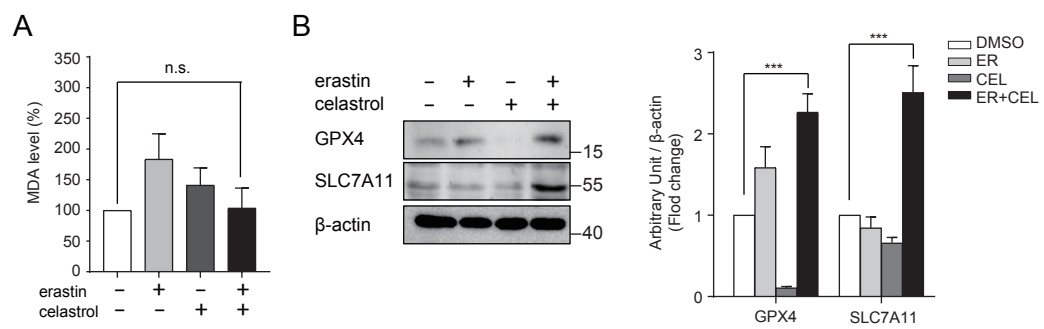

Supplementary Figure 1

Supplement: Supplementary file 1 — Fig. S1. Combination of erastin and celastrol failed to induce ferroptosis. (A) Measurement of cellular MDA in HCC827 cells exposed to erastin and/or celastrol for 24 h. (B) Western blotting analysis of GPX4 and SLC7A11 expression in HCC827 cells. The mean ± SD is shown, n=3. Statistical significance was determined using one‐way ANOVA with Tukey’s post hoc test. ***p<0.001. n.s., not significant. [file MOL2-15-2084-s007.pdf]

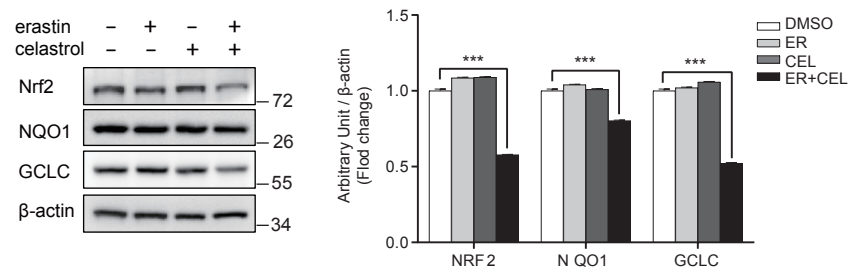

Supplementary Figure 2

Supplement: Supplementary file 2 — Fig. S2. Nrf2, NQO1 and GCLC protein levels in cells exposed to erastin or/and celastrol for 24 h. The mean ± SD is shown, n=3. Statistical significance was determined using two‐way ANOVA with Tukey’s post hoc test. ***p<0.001. [file MOL2-15-2084-s001.pdf]

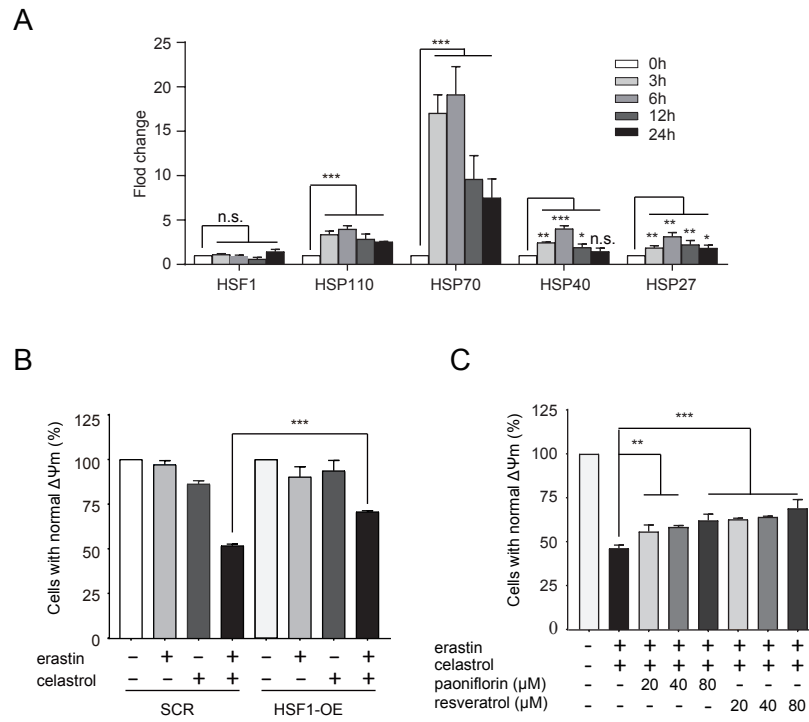

Supplementary Figure 3

Supplement: Supplementary file 3 — Fig. S3. Overexpression or activation of HSF1 inhibited HSP induction and cell death induced by cotreatment with celastrol and erastin. (A) Levels of HSP110, HSP70, HSP40 and HSP27 mRNA in HCC827 cells were examined by RT‐qPCR. HCC827 cells were treated with either erastin or celastrol alone or their combination for the indicated amount of time. (B) HSF1 overexpression prevented cell death induced by cotreatment with celastrol and erastin in HCC827 cells. Cell viability was examined by CCK‐8 assays. (C) CCK‐8 assay demonstrated that activation of HSF1 by paeoniflorin or resveratrol inhibited the cell death induced by cotreatment with celastrol and erastin. The mean ± SD is shown, n=3. Statistical significance was determined using one‐way or two‐way ANOVA with Tukey’s post hoc test. **p<0.01, ***p<0.001. [file MOL2-15-2084-s003.pdf]

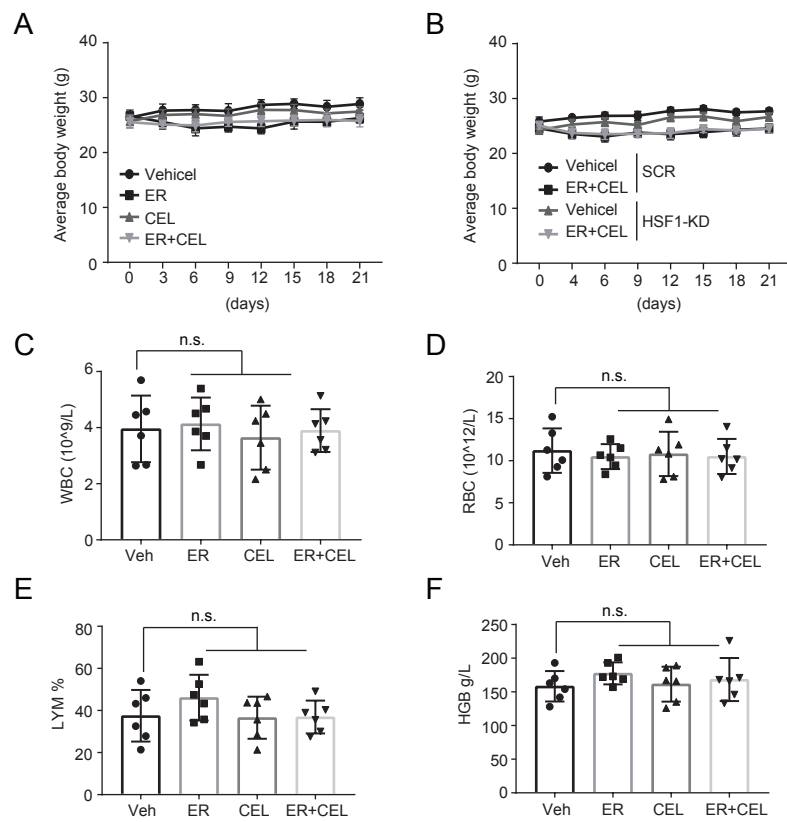

Supplementary Fig. 4

Supplement: Supplementary file 4 — Fig. S4. Changes in body weight and hematologic parameters in mice during treatment. (A, B) Time course (days) of body weight after treatment. (C‐F) Changes of hematologic parameters in mice after treatment with erastin and/or celastrol. Blood samples were collected and the hematologic parameters, including WBC, RBC, LYM and HGB, were examined. The mean ± SD is shown, n=6. Statistical significance was determined using one‐way ANOVA with Tukey’s post hoc test. n.s., not significant. [file MOL2-15-2084-s004.pdf]
